# High-Sensitivity Cardiac Troponin T in Prediction and Diagnosis of Early Postoperative Hypoxemia after Off-Pump Coronary Artery Bypass Grafting

**DOI:** 10.3390/jcdd9120416

**Published:** 2022-11-26

**Authors:** Peng Lu, Xiaohu Lu, Ben Li, Chufan Wang, Xufeng Wang, Yumeng Ji, Zhaoyang Liu, Xiangyu Li, Chenlong Yi, Meijuan Song, Xiaowei Wang

**Affiliations:** 1 Department of Cardiovascular Surgery, The First Affiliated Hospital of Nanjing Medical University, Nanjing 210029, China; 2 Department of Cardiovascular Surgery, The Affiliated Hospital of Yangzhou University, Yangzhou 225000, China; 3 Department of Geriatrics, The First Affiliated Hospital of Nanjing Medical University, Nanjing 210029, China

**Keywords:** Hs-cTnT, hypoxemia, OPCAB, myocardial injury, pulmonary dysfunction

## Abstract

To investigate the relationship of preoperative high-sensitivity cardiac troponin T (hs-cTnT) with early postoperative hypoxemia (EPH) following off-pump coronary artery bypass grafting (OPCAB). Records of patients undergoing OPCAB between 2018 and 2022 were reviewed. Baseline characteristics and postoperative arterial blood gas analysis were derived from the cardiovascular surgery electronic medical records. Preoperative hs-cTnT levels were measured routinely in all patients. Logistic regression analyses were performed to test the association of preoperative hs-cTnT with EPH. A total of 318 OPCAB patients were included, who had a preoperative hs-cTnT test available for review. Before surgery, 198 patients (62%) had a rise in hs-cTnT level (≥14 ng/L) and 127 patients (40%) had a more severe hs-cTnT level (≥25 ng/L). The preoperative hs-cTnT level was associated with EPH (odds ratio per ng/L, 1.86; 95% confidence interval 1.30–2.68; *p* < 0.001), prolonged intensive care unit stay (odds ratio, 1.58; 95% confidence interval 1.08–2.32; *p* = 0.019), and delayed extubating time (odds ratio, 1.63; 95% confidence interval 1.15–2.34; *p* = 0.007). On multivariable analysis, adjusted for BMI, hypertension, smoking status, serum creatinine, and cardiac function, preoperative hs-cTnT remained an independent factor associated with EPH. Elevation of hs-cTnT concentrations are significantly associated with EPH after OPCAB. Review of presurgical hs-cTnT concentration may help identify patients who would benefit from OPCAB to improve surgical risk assessment.

## 1. Introduction

Pulmonary dysfunction has been the most common postoperative complication since the early days of cardiac surgery [1]. The etiology of this pulmonary impairment is believed to be multifactorial, occurring as a result of the combined effects of lung ventilation, surgery, transfusion, and cardiopulmonary bypass (CPB) [2,3,4]. It is recognized that many patients will have altered pulmonary mechanics after cardiac surgery which may appear in a wide range of clinical presentations, from mild hypoxemia to life threatening acute lung injury (ALI) or acute respiratory distress syndrome (ARDS) [5]. Although severe postoperative ARDS is now rare, significant impairment of pulmonary function does occur leading to early postoperative hypoxemia (EPH) and prolonged extubating time [6].

Off-pump coronary artery bypass grafting (OPCAB) is now considered a surgical option to treat multivessel coronary artery disease (CAD) [7]. Regardless of preoperative baseline pulmonary function, respiratory mechanics and gas exchange are strongly affected after OPCAB, increasing the risks of postoperative pulmonary complications. A growing body of evidence suggests that OPCAB cannot confer major protection from postoperative pulmonary dysfunction compared with on-pump coronary artery bypass grafting (CABG) [8,9]. Therefore, developing certain predictive biomarker for respiratory derangement in patients undergoing OPCAB would be of great help for early prediction and diagnosis.

As a specific marker of myocardial injury, high-sensitivity cardiac troponin T (hs-cTnT) is not due to a clinically detectable acute cardiac pathology in most cases. It can be increased in patients without cardiovascular diseases such as non-alcoholic fatty liver disease, chronic obstructive pulmonary disease (COPD), end-stage renal disease, and so on [10,11,12]. Previous studies have found that high presurgical hs-TnT levels were independently predictive of patients developing atrial fibrillation (AF) after cardiac surgery [13]. However, the implications of preoperative hs-cTnT anomalies in the EPH after OPCAB are unknown. We hypothesized that elevated preoperative hs-cTnT concentration can serve as a functional biomarker to assess the risk of EPH after OPCAB.

In this study, we sought to investigate whether preoperative hs-cTnT level is associated with EPH following OPCAB.

## 2. Patients and Methods

### 2.1. Study Design and Population

This study was conducted as a retrospective review with institutional review board approval (2022-SR-403). A total of 318 patients between January 2018 to July 2022 at the Department of Cardiovascular Surgery, Jiangsu Province Hospital, Nanjing Medical University, Nanjing, China was included in this retrospective study. Patients with a Left ventricular ejection fraction (LVEF) ≥25%, left ventricular end-diastolic diameter (LVEDD) ≤70 mm, without diffuse disease in target coronary artery are prone to be performed the revascularization with off pump. All patients operated on by 3 experienced surgeons (WXW, LP, LXH) for OPCAB surgery procedure. The following criteria led to the exclusion of patients: age > 90 years, transfusion, pleural effusions or atelectasis, previous cardiac surgery, primary and secondary myocardiopathy, combination with congenital heart disease, acute coronary syndrome (ACS) reported within 30 days before the surgery, acute heart failure, inflammatory disease or autoimmune disease, infectious or malignant tumor, transplant patients and patients treated with corticosteroids, non-alcoholic fatty liver disease, chronic obstructive pulmonary disease (COPD), end-stage renal disease, valve or combined CABG.

### 2.2. Clinical Data Collection

Clinical data of the enrolled patients collected from medical records was exhaustive, and the following variables were recorded: age, sex, body mass index, smoking status, usual cardiovascular risk factors, previous regular medication, echocardiographic parameters, perioperative laboratory tests, and postoperative arterial blood gas analysis. All of the patients underwent preoperative transthoracic echocardiography (TTE). LVEF was calculated using the Simpson method on the apical four-chamber and apical two-chamber views. EPH was defined as the lowest of the ratio of PaO_2_ to inspired oxygen fraction (PaO_2_/FiO_2_) ≤ 200 mm Hg within 24 h without pleural effusion and pneumothorax [14]. The patients were divided into two groups by early postoperative PaO_2_/FiO_2_: hypoxemia group and non-hypoxemia group.

### 2.3. Anesthesia and Cardiac Surgery Procedure

Patients were pre-medicated with Estazolam orally 12 h before anesthesia. Routine cardiac medications such as β-blockers, angiotensin converting enzyme (ACE) inhibitors, angiotensin receptor blockers (ARB), calcium channel blockers (CCB), and lipid lowering drugs were continued until the morning of the surgery, except for aspirin and clopidogrel, which were stopped at least 1 day earlier. After a radial artery hemodynamic monitoring system was set up in the operating room. Anesthesia was induced with intravenous midazolam (0.03 mg/kg), sufentanil (0.2 to 0.5 mg/kg/h), and propofol (1.5 to 2.5 mg/kg). After verifying that manual ventilation was satisfactory, cisatracurium (0.06 mg/kg/h) was injected. Patients were orally intubated and ventilated with FiO_2_: 40%. Anesthesia was maintained with sufentanil and cisatracurium as required, and inhaled sevoflurane. Following general anesthesia and median sternotomy, the left internal mammary artery (LIMA) and saphenous vein graft were harvested simultaneously in all of the patients. Off-pump surgery procedure was performed on the beating heart utilizing a stabilizer (Medos Biotechnology, Beijing, China) and an intracoronary shunt (Flo-Thru intraluminal Shunt, Synovis Life Technology, Saint Paul, MN, USA). Patients were heparinized intravenously to achieve an activated clotting time >300 s. A side-biting aorta clamp was usually used when proximal anastomoses were performed. Flow measurements are carried out with transit time flow probes (Medtronic, Inc., Minneapolis, MN, USA). After closure of the sternum, patients were transferred safety to the intensive care unit (ICU).

### 2.4. Routine Blood Testing and Serum High-Sensitivity Cardiac Troponin Measurement

Blood samples of the OPCAB patients were collected and analyzed for routine blood testing on the second day morning of hospital admission (Pre-operation) and the second day morning after OPCAB surgery (Post-operation). The following measurements were made: CK, CK-MB, hs-cTnT, N-terminal pro-B-type natriuretic peptide (NT-proBNP), ALT, AST, LDH, hemoglobin, Bun, and creatinine. Biochemical measurements were performed using standard laboratory techniques. Serum hs-cTnT levels were measured using an electrochemiluminescence immunoassay (BML, Inc., Kawagoe, Japan). The lower limit of detection for hs-cTnT was 3 ng/L, and the standard value was below 14 ng/L.

### 2.5. Statistical Analyses

Patients’ characteristics and postoperative outcomes are presented as descriptive statistics. Continuous variables are presented as means ± standard deviations (SD) unless otherwise indicated; categorical variables as numbers (percentages). For continuous data, normality was checked by the Kolmogorov–Smirnov test. Non parametric variables were reported as medians with interquartile ranges (IQR). The means of different hs-cTnT concentrations were compared between patients with EPH and those without EPH using Kolmogorov–Smirnov test. Univariate logistic regression was used to test the association of preoperative hs-cTnT as a continuous variable with major postoperative events. Simple linear regression models were fit to test the associations of patient variables, and operative details in Table 1 with the early lowest PaO_2_/FiO_2_. A double-sided *p*-value < 0.05 was considered statistically significant for all tests. Among the parameters, the following showed significant differences by simple linear regression: BMI, LVEDD, left main coronary disease, NT-proBNP, hs-cTnT. We also added smoking status, hypertension, extent of CAD, serum creatinine and LVEF, as these parameters are clinically significant. Therefore, we performed multivariate, stepwise backward analyses for serum hs-cTnT concentrations. The final model discriminative ability was assessed by calculating the area under the curve (AUC) using a receiver operating characteristics curve and model calibration was assessed with a Hosmer–Lemeshow goodness-of-fit test. All data were analyzed using standard statistical software SPSS version 27.0 (SPSS Inc, Chicago, IL, USA) and GraphPad Prism 9.4 software (GraphPad Software Inc., San Diego, CA, USA).

## 3. Results

### 3.1. Patient Characteristics

A total of 318 patients were included in the analysis, with a mean age of 65.5 ± 8.7 years and a male predominance (74.2%). Demographic and preoperative clinical characteristics for all patients and stratified by presence and absence of EPH are summarized in Table 1. All patients received off-pump coronary artery bypass grafting. The majority of patients (*n* = 241) had three-vessel CAD. The number of grafts was 3.29 ± 0.91. Preoperative LVEF (%) was 60.1 ± 7.7 and the mean EuroSCOREII was 2.2. The body mass index (BMI) was 24.7 ± 2.9 kg/m^2^ in all patients but was significantly higher in the hypoxemia group. During their hospital stay, 107 (33.6%) participants developed EPH.

### 3.2. Correlation between Lowest PaO_2_/FiO_2_ and Baseline Characteristics

**Figure 1:**
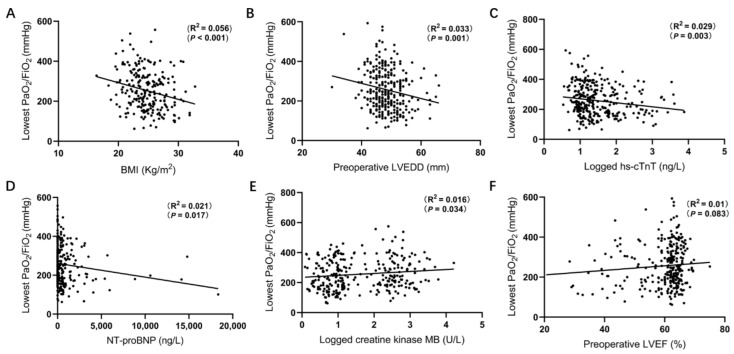
The correlation between lowest postoperative PaO_2_/FiO_2_ levels and baseline characteristics. Postoperative PaO_2_/FiO_2_ was significant correlated with (**A**) BMI (r^2^ = 0.056; *p* < 0.001), (**B**) Preoperative LVEDD (r^2^ = 0.033; *p* = 0.001), (**C**) Hs-cTnT (r^2^ = 0.029; *p* = 0.003), (**D**) NT-proBNP levels (r^2^ = 0.021, *p* = 0.017), (**E**) Creatine kinase MB (r^2^ = 0.016; *p* = 0.034), but not correlated with (**F**) Preoperative LVEF (r^2^ = 0.01; *p* = 0.083).

**Figure 2:**
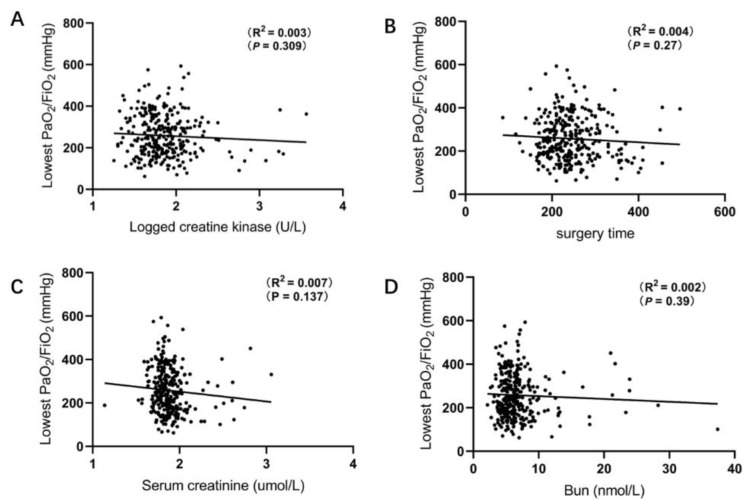
The correlation between lowest postoperative PaO_2_/FiO_2_ levels and baseline characteristics. Postoperative PaO_2_/FiO_2_ was not correlated with (**A**) Creatine kinase (r^2^ = 0.003; *p* = 0.309), (**B**) Surgery time (r^2^ = 0.004; *p* = 0.27), (**C**) Creatinine (r^2^ = 0.007; *p* = 0.137), (**D**) Bun levels (r^2^ = 0.002, *p* = 0.39).

Mean lowest postoperative PaO_2_/FiO_2_ levels were 257.0 ± 101.6 mmHg (range 62–593 mmHg) (Table 2). Associations between laboratory parameters and PaO_2_/FiO_2_ levels were tested using Spearman’s correlation rank test. There was a significant correlation between postoperative PaO_2_/FiO_2_ levels and the BMI (r^2^ = 0.056; *p* < 0.001, Figure 1A), LVEDD (r^2^ = 0.033; *p* = 0.001, Figure 1B), hs-cTnT (r^2^ = 0.029; *p* = 0.003, Figure 1C), NT-proBNP levels (r^2^ = 0.021; *p* = 0.017, Figure 1D), and Creatine kinase MB (r^2^ = 0.016; *p* = 0.034, Figure 1E). In contrast, no significant correlation was observed between postoperative PaO_2_/FiO_2_ levels and the LVEF (r^2^ = 0.01; *p* = 0.083, Figure 1F), Creatine kinase (r^2^ = 0.003; *p* = 0.309, Figure 2A), Surgery time (r^2^ = 0.004; *p* = 0.27, Figure 2B), Creatinine (r^2^ = 0.007; *p* = 0.137, Figure 2C) and Bun (r^2^ = 0.002; *p* = 0.39, Figure 2D).

### 3.3. Cardiac Injury Measurements

Cardiac injury measurements were reviewed for all patients, which were obtained on the second day morning of hospital admission (Pre-operation) and the second day morning after OPCAB surgery (Post-operation). Cardiac injury measurements were determined to be exponentially distributed using a gg-plot and histogram. A total of 198 patients (62%) had an elevated preoperative hs-cTnT that measured ≥14 ng/L, of which 127 patients (40%) had a preoperative hs-cTnT concentration that was higher than 25 ng/L. Mean measurements for the perioperative hs-cTnT, ALT, AST, CK, NT-proBNP, and CK-MB are summarized in Table 1 and Table 3. There was no difference in preoperative LDH, AST, or ALT between patients with and without EPH. However, patients with postoperative hypoxemia were found to have increased preoperative hs-cTnT, NT-proBNP, Creatine kinase, Creatine kinase MB, and Creatinine compared with those without hypoxemia.

### 3.4. Postoperative Events of Patients following OPCAB

The postoperative clinical and laboratory information of the patients enrolled in the study are listed in Table 3. Patients in the hypoxemia group had higher Creatinine, Bun levels but lower ALT concentrations, as shown in Table 3. The hs-cTnT concentration after OPCAB was significantly different between patients with and without EPH (39.62 ng/L; 95% CI; 26.11–83.40 ng/L vs. 16.16 ng/L; 95% CI, 13.85–18.59 ng/L; *p* < 0.001). Postoperative patients in the non-hypoxemia group necessitated shorter intensive care unit, extubated time, and hospital stay. A summary of postoperative arterial blood gas parameters and preoperative hs-cTnT concentrations is presented in Table 2. The majority of patients (*n* = 198) had an elevated hs-cTnT concentration higher than 14 ng/L. The patients in elevated hs-cTnT group received a lower PaO_2_ and PaO_2_/FiO_2_ level, but there was no significant difference in PaCO_2_ and SaO_2_ between two hs-cTnT groups.

### 3.5. Association of Preoperative hs-cTnT with EPH

**Figure 3:**
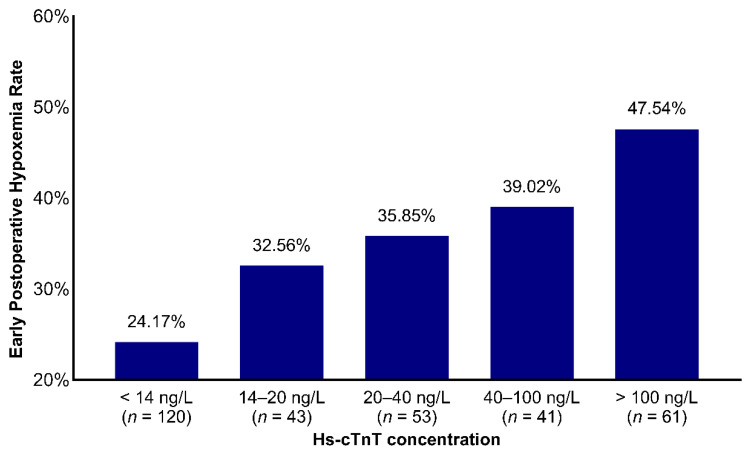
The association of preoperative hs-cTnT levels with EPH after OPCAB demonstrated a stepwise increase in rates with early postoperative hypoxemia (Mantel–Haenszel test for trend *p* = 0.029). Using presurgical hs-cTnT <14ng/L as a reference, the odds ratio for hypoxemia were 0.66 (95% confidence interval, 0.32–1.37) for hs-cTnT 14–20 ng/L; 0.57 (95% confidence interval, 0.29–1.19) for hs-cTnT 20–40 ng/L, 1.50 (95% confidence interval, 0.24–1.09) for hs-cTnT 40–100 ng/L, and 0.35 (95% confidence interval, 0.19–0.68) for hs-cTnT >100 ng/L.

EPH was recorded in 33.6% of patients (Table 4). The rate of early postoperative hypoxemia following OPCAB increased gradually for patients with high hs-cTnT level, as depicted in Figure 3. EPH rate ranged from 24.17% to 47.54% for patients with hs-cTnT < 14 ng/L and >100 ng/L, respectively (Mantel–Haenszel test for trend *p* = 0.029). Postoperative stroke (overall, 2.2%) were rare, and not associated with preoperative hs-cTnT levels (Table 4). In addition to EPH, preoperative hs-cTnT concentration was also associated with prolonged length of intensive care unit stay and delayed extubated time (Table 4).

### 3.6. Factors Associated with EPH

**Figure 4:**
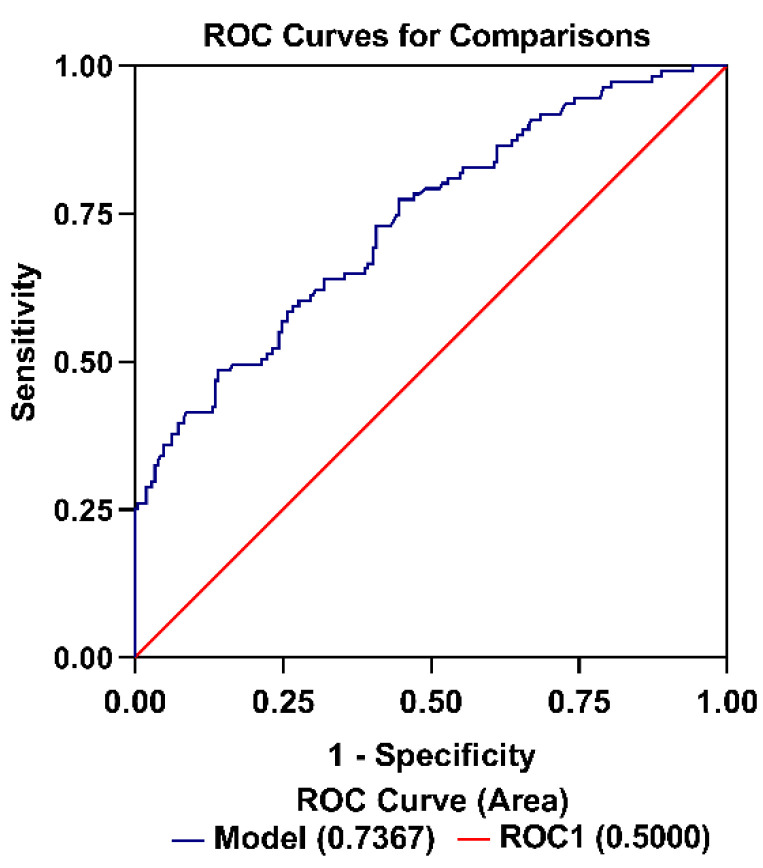
Receiver operating characteristic (ROC) curve showing sensitivity and 1-specificity for the model used to assess EPH rate from presurgical hs-cTnT level. Multivariable model area under the curve (AUC) was 0.7367 (95% confidence interval, 0.679–0.795; Hosmer–Lemeshow goodness-of-fit test *p* = 0.025).

Univariate analyses were performed to identify patient and clinical factors associated with EPH that may confound the relationship with hs-cTnT, which were then included in the multivariable model. We found that hs-cTnT ≥ 14ng/L concentration increased the odds of hypoxemia (odds ratio [OR], 0.42; 95% CI, 0.22–0.80; *p* = 0.01) independent of other factors on multivariable analysis (Table 5). The receiver operating characteristics for hs-cTnT to discriminate hypoxemia in the multivariable model is depicted in Figure 4. The multivariable model AUC for hs-cTnT was 0.7367 (95% CI, 0.679–0.795; Hosmer–Lemeshow goodness-of-fit test *p* = 0.025).

## 4. Discussion

This single institution retrospective cohort study examines the association of preoperative hs-cTnT with EPH following OPCAB. Our results showed that elevated hs-cTnT concentration was significantly associated with an increased risk of EPH and prolonged hospital stay after OPCAB, independent of BMI, smoking status, serum creatinine, extent of CAD, and cardiac function. This study is important because it shows that perioperative risk assessment for CABG may be further improved by presurgical hs-cTnT tests, and may help identify patients who benefit from lung protection strategies.

Hypoxemia is a serious complication of cardiac surgery, and it is associated with increased hospital length of stay, morbidity, and mortality [15,16]. Even when ALI is avoided, milder forms of hypoxemia are associated with adverse outcomes and degree of hypoxemia correlates negatively with long-term survival despite successful hospital discharge. Pulmonary hypoxemia after cardiac surgery is multifactorial in origin, and there are multiple risk factors that contribute to the development of respiratory dysfunction. Avoiding excessive perioperative transfusions, improving extracorporeal techniques and the optimization of ventilation, and hemodynamics seem to be the most modifiable risk factors. Symptoms and signs of acute lung injury, including cough, expectoration, and asthma, may occur after CABG, and may cause respiratory failure [17]. Therefore, protecting the lungs from hypoxemia throughout CABG procedure is becoming a focus of particular interest among cardiovascular surgeons. Our study is performed on patients who underwent OPCAB to determine if preoperative hs-cTnT is a functional biomarker for postoperative hypoxemia in this form of cardiac surgery. Preoperative hs-cTnT has a good sensitivity and specificity for diagnosis of EPH after OPCAB, and can be used as an early biomarker for prediction of postoperative hypoxemia.

Myocardial ischemia and reperfusion (I/R) occur in cardiac surgery and myocardial infarction (MI) receiving OPCAB [18]. Ischemia followed by reperfusion, in coronary artery beds, can lead to distant lung injury. This type of injury affects the alveolar epithelium and the capillary endothelium. Studies in humans and rabbits showed that both types of cells have features compatible with apoptosis and necrosis [19,20]. In clinical practice, a sharp rise in serum hs-cTnT and creatine kinase MB has been regarded as a sign of acute myocardial infarction. High sensitivity cardiac troponin T (hs-cTnT) and creatine kinase MB has traditionally been tested for the severity of myocardial injury throughout cardiac surgery. Reperfusion injury in the ischemic myocardium following OPCAB is accompanied with the elevated hs-cTnT concentration in circulation [21,22]. In this study, we confirmed that elevated preoperative hs-cTnT level is associated with EPH following OPCAB. Postoperative hypoxemia may also be associated with the severity of ischemia and reperfusion injury at distant, myocardial sites. In spite of its widespread use in assessing myocardial injury, hs-cTnT may function as an important functional biomarker in postoperative ALI.

OPCAB is part of the procedural armamentarium of a growing proportion of cardiac surgeons worldwide, especially in Asia. The continuous effort to innovate, adapt, improve clinical outcomes, and optimize patient care at our institution has led to a shift toward OPCAB in the treatment of patients undergoing CAD, reaching an 84% of CABG operations in 2018 and continuously 95% since 2019. In the general population, OPCAB has been associated with similar short-term outcomes, at least when performed by experienced surgeons. In the long term, inferior outcomes have been reported with OPCAB [23]. Studies comparing patients between OPCAB and on-pump CABG remains debated. It has been shown that OPCAB cannot confer major protection from postoperative pulmonary dysfunction compared with on-pump CABG. Postoperative pulmonary dysfunction is multifactorial in origin but seems to be irrelevant with the use of CPB. Our results demonstrate that preoperative hs-cTnT is strongly associated with EPH following OPCAB. Further research should examine if elevated hs-cTnT is associated with EPH after on-pump CABG and if elevated hs-cTnT identifies EPH complication following other non-cardiac surgery.

However, this is a nonrandomized retrospective study. Therefore, this study has several limitations. Due to the retrospective study design, residual bias, and unconsidered confounding factors may have contributed to our findings. Furthermore, we acknowledge that we did not stratify our patients by SYNTAX score. We were also unable to rule out the possibility that postoperative pulmonary dysfunction may have been due to concomitant mechanical ventilation, which have proved to have been important factors in influencing respiratory function [24]. In addition, we did not have control over arterial blood gas timepoint, we chose to select the lowest of the ratio of PaO_2_/FiO_2_ after OPCAB. Prospective study including seven arterial blood sampling timepoints has already been under investigation in our center.

## 5. Conclusions

In this single center cohort study, we demonstrate that preoperative hs-cTnT concentration is associated with increased risk of EPH after OPCAB. Preoperative hs-cTnT may enhance perioperative risk assessment of postoperative hypoxemia independent of other known cardiopulmonary risk factors. Preoperative assessment of hs-cTnT concentration should be considered for patients prone to postoperative pulmonary dysfunction.

## Figures and Tables

**Table 1:** Demographic and clinical characteristics of patients undergoing OPCAB.

Characteristics	ALL (*n* = 318)	Hypoxemia (*n* = 107)	Non-Hypoxemia (*n* = 211)	*p* Value
Age (y)	65.5 ± 8.7	65.3 ± 8.8	65.7 ± 8.5	0.68
Gender				
Female	82 (25.8)	23 (21.5)	59 (28.0)	0.21
Male	236 (74.2)	84 (78.5)	152 (72.0)	
BMI (kg/m^2^)	24.7 ± 2.9	25.4 ± 2.6	24.4 ± 3.0	0.013
Smoking status				
Never smoked	168 (52.8)	48 (44.9)	120 (56.9)	0.079
Past smoker	86 (27.0)	31 (30.0)	55 (26.1)	
Current smoker	64 (20.1)	28 (26.2)	36 (17.1)	
Diabetes mellitus	107 (33.6)	38 (35.5)	69 (32.7)	0.616
Hypertension	217 (68.2)	78 (72.9)	139 (65.9)	0.25
Hyperlipidemia	39 (12.3)	12 (11.2)	27 (12.8)	0.685
Preoperative LVEF (%)	60.1 ± 7.7	59.3 ± 8.3	60.4 ± 7.3	0.21
Preoperative LVEDD, mm	48.3 ± 4.8	49.1 ± 4.0	48.0 ± 5.1	0.082
EuroSCOREII	2.2 ± 0.5	2.3 ± 0.4	2.2 ± 0.7	0.692
Prior myocardial infarction	96 (30.2)	31 (29.0)	65 (30.8)	0.736
Peripheral vascular disease	33 (10.4)	12 (11.2)	21 (10.0)	0.727
Previous treatments				
Aspirin	298 (93.7)	97 (90.7)	201 (95.3)	0.109
Beta blockers	238 (74.8)	82 (76.6)	156 (73.9)	0.6
Statins	216 (67.9)	72 (67.3)	144 (68.2)	0.863
ACE inhibitors	221 (69.5)	75 (70.1)	146 (69.2)	0.87
CCS	2 (2–3)	2 (2–3)	2 (2–3)	0.79
NYHA	2 (2–3)	2 (2–3)	2 (2–3)	0.578
Left main coronary disease	65 (20.4)	30 (28.0)	35 (16.6)	0.017
Preoperative biological data				
Serum creatinine, umol/L	71.2 (61.0–86.0)	76.0 (64.6–94.1)	69.1 (59.6–83.2)	0.002
Bun, mmol/L	6.6 ± 3.6	6.8 ± 4.1	6.6 ± 3.4	0.52
Haemoglobin, g/L	129.3 ± 19.3	130.3 ± 18.1	128.9 ± 19.7	0.24
AST, U/L	35.7 ± 34.6	32.48 ± 24.4	37.3 ± 38.6	0.41
ALT, U/L	40.2 ± 38.4	37.7 ± 27.7	41.5 ± 42.9	0.93
LDH, U/L	199 (175–229.2)	201 (175–265)	197 (174.5–222)	0.11
Creatine kinase, U/L	65 (46.5–96.5)	68 (47.2–103.5)	63 (46–93)	0.014
Creatine kinase MB, U/L	14.4 (5.6–316.92)	11.7 (4.6–228.3)	23.2 (6.8–366.6)	0.033
NT-proBNP, ng/L	200.9 (1.88–652.5)	393.9 (2.53–904.5)	115 (1.79–549)	0.007
Hs-cTnT, ng/L	19.77 (10.42–59.53)	39.62 (18.1–287)	16.16 (9.09–30.33)	<0.001
Extent of CAD				
1 VD	26 (8.2)	8 (7.5)	18 (8.5)	0.46
2 VD	51 (20.1)	21 (19.6)	30 (14.2)	
3 VD	241 (75.8)	78 (72.9)	163 (77.3)	

Values are presented as mean ± standard deviation, median (interquartile range) or *n* (%). OPCAB, off-pump coronary artery bypass; BMI, body mass index; LVEF, left ventricular ejection fraction; LVEDD, left ventricular end-diastolic dimension; EuroSCORE II, European System for Cardiac Operative Risk Evaluation II; CCS, Canadian classification score for angina grade; NYHA, New York Heart Association grade for heart failure; CAD, coronary artery disease.

**Table 2:** Lowest postoperative arterial blood gas parameters relative to hs-cTnT following OPCAB.

Variables	ALL (*n* = 318)	Hs-cTnT ≥ 14 (*n* = 198)	Hs-cTnT < 14 (*n* = 120)	*p* Value
PaO_2_ (mm Hg)	126.1 ± 50.74	120.6 ± 42.1	135.6 ± 61.94	* 0.012
PaCO_2_ (mm Hg)	38.05 ± 4.98	37.91 ± 5.23	38.28 ± 4.53	0.53
PaO_2_/FiO_2_ Ratio (mm Hg)	257.0 ± 101.6	247.1 ± 95.64	283.3 ± 124.6	* 0.005
SaO_2_ (%)	97.33 ± 6.87	97.45 ± 4.2	97.12 ± 9.92	0.684

Values are presented as mean ± standard deviation. * Significant finding with *p* < 0.05.

**Table 3:** Postoperative clinical and laboratory information of patients undergoing OPCAB.

Characteristics	ALL (*n* = 318)	Hypoxemia (*n* = 107)	Non-Hypoxemia (*n* = 211)	*p* Value
**In-hospital stay after surgery (days)**	8.5 ± 2.1	8.7 ± 1.7	8.1 ± 2.6	0.009
ICU stay (days)	2.7 ± 1.5	2.8 ± 1.4	2.4 ± 1.6	0.008
Extubated time (days)	1.5 ± 1.0	1.6 ± 1.1	1.3 ± 0.6	0.021
30-day mortality	5 (1.6)	2 (1.9)	3 (1.4)	0.762
Postoperative stroke	7 (2.2)	2 (1.9)	5 (2.4)	0.774
Postoperative AF	42 (13.21)	14 (13.08)	28 (13.27)	0.963
Deep sternal wound infection	15 (4.7)	6 (5.6)	9 (4.3)	0.594
Hs-cTnT, ng/L	132 (85–244)	152 (91–278.5)	124 (83.5–203.2)	0.028
NT-proBNP, ng/L	920 (420–1863)	997.5 (427.7–1943.2)	877 (420–1807)	0.62
Creatine kinase, U/L	325 (211–529)	355 (214–573.5)	313 (205–502)	0.25
Creatine kinase MB, U/L	6 (4–11)	6 (3–10)	6 (4–11)	0.45
AST, U/L	39.74 ± 29.17	35.57 ± 19.1	41.8 ± 32.88	0.068
ALT, U/L	32.77 ± 29.64	27.34 ± 19.66	35.34 ± 33.21	0.019
LDH, U/L	309 ± 91.24	317.9 ± 90.43	304.2 ± 91.56	0.227
Serum creatinine, umol/L	77.2 (63.08–99.53)	85.4 (69.2–110.6)	75.1 (62.1–91.5)	<0.001
Bun, mmol/L	6.7 ± 3.59	7.39 ± 4.4	6.37 ± 3.1	0.015
Haemoglobin, g/L	109.3 ± 16.79	110.2 ± 17.63	108.9 ± 16.38	0.52

Values are presented as mean ± standard deviation, median (interquartile range), or *n* (%). OPCAB, off-pump coronary artery bypass; ICU, Intensive care unit; AF, atrial fibrillation.

**Table 4:** Association of postoperative events with preoperative hs-cTnT levels (logged).

Variable	Patients with Event (*n* = 318)	Crude Odds Ratio hs-cTnT (Per ng/L)	95% Confidence Interval	*p* Value
**EPH**	107 (33.6)	1.86	(1.30–2.68)	<0.001
30-day mortality	5 (1.6)	0.73	(0.10–2.69)	0.683
Postoperative AF	42 (13.2)	1.17	(0.70–1.86)	0.541
Postoperative stroke	7 (2.2)	0.90	(0.21–2.60)	0.866
Deep sternal wound infection	15 (4.7)	0.72	(0.25–1.64)	0.465
Length of In-hospital stay after surgery > 9 d	94 (29.6)	1.42	(0.98–2.04)	0.065
ICU stay > 3 d	97 (30.5)	1.58	(1.08–2.32)	0.019
Extubated time > 1 d	90 (28.3)	1.63	(1.15–2.34)	0.007

Values are presented as *n* (%) unless otherwise indicated. AF, atrial fibrillation; EPH, early postoperative hypoxemia; ICU, Intensive care unit.

**Table 5:** Multivariable logistic regression model for early postoperative hypoxemia after OPCAB.

Effect	Ajusted Odd Ratio	95% Wald Confidence Limits	*p* Value
Preoperative hs-cTnT			
<14 ng/L	Reference		
≥14 ng/L	0.45	0.23–0.86	0.016
Smoking status			
Never smoked	Reference		
Past smoker	0.84	0.41–1.72	0.64
Current smoker	1.24	0.55–2.77	0.59
BMI			
<24 kg/m^2^	Reference		
≥24 kg/m^2^	2.66	1.35–5.41	0.006
Hypertension	0.96	0.49–1.85	0.90
NT-proBNP			
≤100 ng/L	Reference		
>100 ng/L	1.06	0.54–2.10	0.87
Left main coronary disease	0.66	0.33–1.36	0.257
Extent of CAD			
1 VD	Reference		
2 VD	2.16	0.61–8.29	0.244
3 VD	1.11	0.37–3.60	0.86
Preoperative creatinine			
≤133 umol/L	Reference		
>133 umol/L	0.8	0.19–2.92	0.745
Preoperative LVEF (per %)	0.96	0.91–1.0	0.098
Preoperative LVEDD (per mm)	1.00	0.92–1.08	0.938

BMI, body mass index; CAD, coronary artery disease; LVEF, left ventricular ejection fraction; LVEDD, left ventricular end-diastolic dimension.

## Data Availability

The original contributions presented in the study are included in the article, and further inquiries can be directed to the corresponding author.

## Abbreviations and Acronyms

ACS	acute coronary syndrome
ALI	acute lung injury
ARDS	acute respiratory distress syndrome
BMI	body mass index
CABG	coronary artery bypass grafting
CAD	coronary artery disease
CPB	cardiopulmonary bypass
COPD	chronic obstructive pulmonary disease
EPH	early postoperative hypoxemia
Hs-cTnT	high-sensitivity cardiac troponin T
ICU	intensive care unit
I/R	ischemia and reperfusion
LVEDD	left ventricular end-diastolic diameter
LVEF	Left ventricular ejection fraction
LIMA	left internal mammary artery
MI NT-proBNP	myocardial infarction N-terminal pro-B-type natriuretic peptide
OPCAB	off-pump coronary artery bypass grafting
TTE	transthoracic echocardiography
